# Sanguiins—Promising Molecules with Broad Biological Potential

**DOI:** 10.3390/ijms222312972

**Published:** 2021-11-30

**Authors:** Jakub Gesek, Katarzyna Jakimiuk, Atanas G. Atanasov, Michał Tomczyk

**Affiliations:** 1 Student’s Scientific Association, Department of Pharmacognosy, Faculty of Pharmacy with the Division of Laboratory Medicine, Medical University of Białystok, ul. Mickiewicza 2a, 15-230 Białystok, Poland; jgesek1@student.umb.edu.pl; 2 Department of Pharmacognosy, Faculty of Pharmacy with the Division of Laboratory Medicine, Medical University of Białystok, ul. Mickiewicza 2a, 15-230 Białystok, Poland; katarzyna.jakimiuk@umb.edu.pl; 3 Ludwig Boltzmann Institute for Digital Health and Patient Safety, Medical University of Vienna, Spitalgasse 23, 1090 Vienna, Austria; atanas.atanasov@dhps.lbg.ac.at; 4 Institute of Genetics and Animal Biotechnology, Polish Academy of Sciences, Jastrzębiec, 05-552 Magdalenka, Poland; 5 Department of Pharmaceutical Sciences, University of Vienna, Althanstrasse 14, 1090 Vienna, Austria

**Keywords:** sanguiin, biological activity, ellagitannins, Rosaceae

## Abstract

Compounds of natural origin, an infinite treasure of bioactive chemical entities, persist as an inexhaustible resource for discovering new medicines. In this review, we summarize the naturally occurring ellagitannins, sanguiins, which are bioactive constituents of various traditional medicinal plants, especially from the Rosaceae family. In-depth studies of sanguiin H-6 as an antimicrobial, antiviral, anticancer, anti-inflammatory, and osteoclastogenesis inhibitory agent have led to potent drug candidates. In addition, recently, virtual screening studies have suggested that sanguiin H-6 might increase resistance toward SARS-CoV-2 in the early stages of infection. Further experimental investigations on ADMET (absorption, distribution, metabolism, excretion, and toxicity) supplemented with molecular docking and molecular dynamics simulation are still needed to fully understand sanguiins’ mechanism of action. In sum, sanguiins appear to be promising compounds for additional studies, especially for their application in therapies for a multitude of common and debilitating ailments.

## 1. Introduction

Most of the discovered drugs are either drugs of natural origin or synthetic derivatives of natural compounds. Thus, a multidisciplinary approach to drug discovery and molecular diversity from natural product sources needs to be combined to provide the best solution to the problems with drug discovery and development [1,2]. Plants are known to be a rich source of pharmacologically active secondary metabolites divided into structural chemical classes [3,4]. One of the pharmacologically valuable classes of phytoconstituents are ellagitannins (ETs), and belonging to them, sanguiins. ETs, water-soluble phenolics, are esters of hexahydroxydiphenic acid and a polyol, usually β-D-glucose or quinic acid [5,6,7]. ET compounds demonstrate an enormous structural variability connected with various possibilities for the linkage of hexahydroxydiphenic residues with the glucose moiety and particularly by their easy susceptibility to creating dimeric and oligomeric derivatives [8]. The polyphenol-protein system and its interactions may underlie the medicinal properties exhibited by members of the ETs family. Fruits and nuts are rich sources of ellagitannins and are important in the human diet due to their properties as micronutrients [9,10]. Due to the limited bioavailability of ellagitannins, as orally administered and the metabolic chemical changes as a result of their transit through the gastrointestinal tract, comprising of hydrolysis and gut microbiota metabolism, the activity of the produced metabolites also needs to be taken into consideration [3].

Sanguiins, members of the ET class of hydrolyzable plant polyphenols, are found mainly in the Rosaceae family and are primarily widespread in berries. The main advantage of sanguiins over other common polyphenols in the plant world is their wide distribution in food products. Therefore, their health-promoting properties can be used in a properly balanced diet [11]. In addition to the natural occurrence of sanguiin, there are reports on the synthetic production of sanguiin H-5 [12]. The structural features of sanguiins make them a demanding molecular target. Sanguiin H-1 comprises the characteristic hexahydrodiphenoyl (HHDP) moiety linked with β-D-glucose and 1,6-di-*O*-galloyl moieties. On the other hand, sanguiin H-2 possesses one galloyl moiety and two sanguisorboyl linking ester groups. Comparing the H-1 and H-4 sanguiins, they differ only in an additional galloyl substituent in sanguiin H-1. The structure of sanguiin H-3, a dimeric ellagitannin, contains two glucose substitutions. Furthermore, the complex structure of sanguiin H-6 includes sanguiin H-2 and pedunculagin moieties. The chemical structure of sanguiin H-10 closely resembles sanguiin H-2, except sanguiin H-10 contains an extra HHDP group. Substitution patterns of sanguiin H-11 also show similarities to sanguiin H-2. The only difference between these structures is the lack of a galloyl moiety in the sanguiin H-11 [13].

Although various bioactivities (e.g., antioxidant, anticancer, antiviral, and antimicrobial) of sanguiins, mainly sanguiin H-6, have been investigated, their pharmacological potential demonstrated in vitro, in silico, and in vivo experimental models has not been clearly organized through review articles. Thus, this manuscript summarizes the findings on the widespread bioactivities of sanguiin H-1 (SH1), sanguiin H-2 (SH2), sanguiin H-3 (SH3), sanguiin H-4 (SH4), sanguiin H-6 (SH6), sanguiin H-10 (SH10), and sanguiin H-11 (SH11) to showcase their potential to be used as therapeutic agents.

## 2. Methodology

A broad search strategy was used to find English language publications indexed in SCOPUS, PubMed/MEDLINE, Google Scholar, Web of Science (SCI-EXPANDED), Wiley Online Library, Taylor & Francis Online, REAXYS Database, Science Direct/ELSEVIER, and EBSCO Discovery Service (EDS) [14]. These databases were searched systematically for articles published from 1982 to 2021. Relevant publications were selected manually from the following searches: sanguiin, sanguiins, Rosaceae, traditional use, traditional medicine, folk medicine, sanguiin H-6, sanguiin H6, sanguiin H-10, sanguiin H10, sanguiin H-5, sanguiin H5, sanguiin H-2, sanguiin H2, sanguiin H-11, sanguiin H11, sanguiin H-4, sanguiin H4, sanguiin H-3, sanguiin H3, ellagitannins, tannins, *Rubus*, anticancer, antiviral, SARS-CoV-2, COVID-19, antioxidant, anti-inflammatory, biological activity, antimicrobial, biological potential, metabolism, clinical trials, preclinical trials, chemistry, galloyl moiety, absorption, distribution, excretion, toxicity, perspectives, trials, pharmacological, natural product, secondary metabolites, therapeutic agent, inhibitory activity, inhibitors, dose, efficacy, exposure, experimental model, quantitative analysis, qualitative analysis, geographical location, as well as each of species containing sanguiins combined with traditional use, traditional medicine, or folk medicine. The search terms operated in separate or limited combinations that considered the requirements or limitations of the database being used.

## 3. Natural Occurrence of Sanguiins

**Figure 1:**
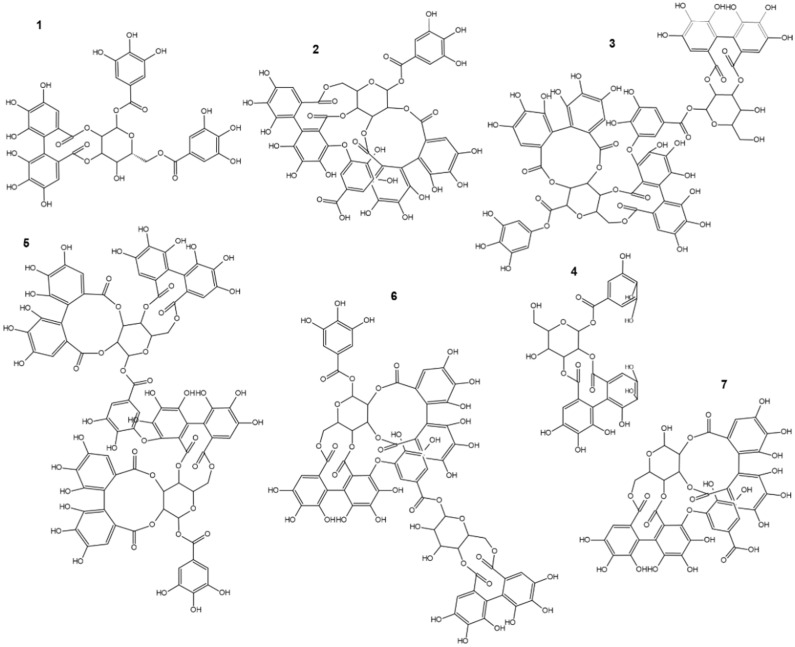
Sanguiins presented in natural sources. (**1**) Sanguiin H-1, (**2**) sanguiin H-2, (**3**) sanguiin H-3, (**4**) sanguiin H-4, (**5**) sanguiin H-6, (**6**) sanguiin H-10, and (**7**) sanguiin H-11.

Among various phenolic compounds isolated from the Rosaceae family, tannins and related compounds seem to have a leading position. It is known that plants previously used in folk medicine represent a suitable beginning to discover new potent drugs to treat various human disorders [15]. Sanguiins (Figure 1), naturally occurring ET, have been isolated chiefly from *Rubus* species and are used as a traditional drug to cure, e.g., diarrhea, menstrual pain, menopause disorders, liver diseases, aphtha, gingivitis, as well as fever, angina, enteritis, hepatitis, concretion, eczema, rheumatism, enterocolitis, bronchitis, prostate disorders, pain, cold, cough, and fever (Table 1) [16,17]. Moreover, SH6 seems to be the most widespread within plants of the *Rubus* and is present in 22 species of this genus. Furthermore, the largest number of isolated and identified types of sanguiins, including SH2, SH4, SH5, SH6, and SH11, are found in *Rubus coreanus* [18]. Besides the *Rubus* genus, sanguiins and their isomers are found and reported in *Alchemilla vulgaris*, *Alchemilla mollis* [19], *Duchesnea indica* [20], *Euphorbia fischeriana* [21], *Fragaria vesca*, *Fragaria ananassa* [22], *Punica granatum* [23], *Terminalia calamansanai* [24], as well as in *Sanguisorba officinalis* [25], and *Sanguisorba tenuijolia* var. *alba* [18].

Among all sanguiins detected in plant material, only part of them was quantitatively analyzed. The place of harvest displays a relevant role in the amount of isolated sanguiins. For example, in *Rubus fruticosus* fruits, the range of detected SH6 is 135.04–547.48 mg/100 g of d.w. (dry weight) [26] and in *Rubus idaeus* shoots, 170.9–633.1 mg/100 g of d.w of the extract [27]. Following that, sanguiins content depends on fruits’ ripeness, harvest time, climate, geographic location, and mineral nutrition [10,28]. It is reported that in *Rubus* and *Fragaria* species, ellagitannins content represents a range of 50% to 80% of all phenolic compounds [10,29]. In this review, the list of plants that produce sanguiins and their reported traditional uses are tabulated in Table 1.

## 4. Chromatographic Techniques for the Analysis of Sanguiins

Chromatography displays a crucial role in the analysis of chemical compound mixtures. As a method for the separation and analysis of extracts and fractions from plants, it provides the possibility of qualitative and quantitative determination of the test substance with high resolution [67]. Chromatographic techniques and analysis conditions for detection, quantitative determination, and isolation of sanguiins and their isomers are given in Table 2.

## 5. Biological Potential of Sanguiins

Sanguiins, as one of the subgroups of polyphenolic ellagitannins, exhibit various pharmacological activities due to having different chemical structures. They possess a broad spectrum of pharmacological features such as anticancer, anti-inflammatory, antioxidant, osteoprotective, estrogenic, antibacterial, antifungal, and antiviral (including SARS-CoV-2), as shown in Table 3. Various in vivo and in vitro investigations on sanguiins, especially on sanguiin H-6, have elucidated their medicinal characteristics and mechanisms of action [68,69].

### 5.1. Antioxidant and Anti-Inflammatory Activities

One of the best-shown properties of polyphenols, and following that, sanguiins, is the potential antioxidant effect. Most references mention sanguiin H-6 as the primary compound having antioxidant activity, e.g., its influences on stress and oxidative damage were investigated. The production of peroxynitrite (ONOO-) was induced by the administration of lipopolysaccharide (LPS), followed by the induction of ischemia and reperfusion [88]. It was revealed that receiving SH6 before induction of oxidative damage could reduce the adverse effects associated with the release of ONOO- and enhance the improvement of injured kidney function [72]. Another chemical compound belonging to the sanguiins group that exhibits antioxidant activity is SH11. An examination of the protective effect of SH11 isolated from *Sanguisorbae radix* and its mechanism against glutamate-induced death in HT22 murine hippocampal cells exposed a significant reduction in glutamine-induced reactive oxygen radicals’ accumulation and calcium ion influx [74]. Furthermore, ellagitannins from the berries of the *Rubus* family, including dimeric SH6 and SH10, function both as radical scavengers (in a DPPH test) and as antioxidants toward lipid oxidation in food emulsions (studied in bulk and emulsified methyl linoleate, in human low-density lipoprotein in vitro) [75]. The impact of sanguiins on the inflammation process was investigated by measuring their effect on rat neutrophils’ chemotaxis. SH11 and SH6 effectively inhibited the cytokine-induced neutrophil chemoattractant migration process by 10.7% and 33%, respectively, in comparison with the control. Additionally, the study showed no toxic effect of sanguiin on neutrophils [70]. Furthermore, at a concentration of 2.5 μM, SH6 completely inhibited the release of IL-8 induced by tumor necrosis factor α and interleukin-1β and inhibited TNFα stimulated NF-κB transcription [71]. SH6 caused a concentration-dependent reduction in nitrite production, regression in induced NO synthase (iNOS) activity, and an increase in cell viability. Moreover, SH6 showed an apparent scavenging effect for NO generated from sodium nitroprusside (NO donor) [76].

### 5.2. Osteoclastogenesis Inhibitory Activity

In a subsequent in vitro study, the action of *Rubus parvifolius* L. and its main component, SH6, was tested as the inhibitor of osteoclastogenesis and bone resorption. Sanguiin influence was based on the reduction in osteoclast differentiation and bone resorption, a decrease in the production of reactive oxygen species, as well as the inhibition of the nuclear translocation of the nuclear factor of activated T cells cytoplasmic-1 (NFATc1), c-Fos, and nuclear factor-κB. Additionally, sanguiin reduced the levels of NFATc1, cathepsin K, c-Src, and inhibited in vivo TNF-α-mediated osteoclastogenesis [47].

### 5.3. Antibacterial Activity

The growing resistance of bacteria to currently used antibiotics is a growing problem in current medicine [89]. Increasingly emerging research on sanguine antibacterial properties gives hope for the discovery of antibacterial agents with the lack of unpleasant side effects. Examination of the antibacterial activity of fruits of selected *Rubus* species and compounds (SH6 and ellagic acid) against selected Gram-negative and Gram-positive bacteria allowed assessment of their usefulness in the fight against microorganisms. The results showed that SH6 was active against *Streptococcus A* (MIC = 0.5 mg/mL), *Streptococcus pneumoniae* (MIC = 0.5 mg/mL), *Corynebacterium diphtheriae* (MIC = 0.03 mg/mL), *Bacillus subtilis* (MIC = 0.5 mg/mL), *Clostridium sporogenes* (MIC = 0.06 mg/mL), *Staphylococcus aureus* (MIC = 0.25 mg/mL), *Staphylococcus epidermidis* (MIC = 0.125 mg/mL), and *Moraxella catarrhalis* (MIC = 0.5 mg/mL) [27].

Additionally, another study showed that SH6 exhibited a significant inhibition level against *S. aureus*, *E. coli*, and *C. perfringens* [77]. *Rubus ulmifolius* fruit extract containing SH10, showed an antibacterial effect against *Escherichia coli*, *Morganella morganii*, and *Proteus mirabilis*, but higher extract concentrations were required: MIC = 5 mg/mL, MIC = 5 mg/mL, and MIC = 10 mg/mL, respectively [78].

### 5.4. Antifungal Activity

Moreover, *Rubus ulmifolius* fruit extract was tested as an antifungal agent. It was proved that the extract containing SH6 exhibited fungistatic activity against *Candida albicans*. The minimum inhibitory concentration was 5 mg/mL. Unfortunately, the extract did not show any fungicidal activity, achieving a result of >20 mg/mL [78].

### 5.5. Antiviral Activity (Including SARS-CoV-2)

Viruses, as pathogenic microorganisms, show significant genetic variability and the ability to mutate. Often, they do not show signs of infection at first. Currently, an increasing number of drug-resistant strains, as well as the toxicity of previously known drugs, force researchers to develop new antiviral substances [90]. In recent months, the entire world has been severely affected by the SARS-CoV-2 pandemic, which has led scientists to focus their attention on potential candidates against its eradication. More and more recent research conducted worldwide shows that sanguiins may be a potential candidate in the fight against viral diseases, including COVID-19 [91,92]. One of the studies predicted that SH6 is a compound that binds very well to the S1 and S2 subunits of the SARS-CoV-2 virus spine, which is responsible for entering the host cells and causing infection. SH6 showed the best binding energy among all tested compounds in the molecular docking assay. Additionally, SH2, also mentioned in the study, showed a lower result than the one mentioned above. Moreover, sanguiin has been proposed to act not only against the spike subunits of the SARS-CoV-2 virus [93]. Another molecular docking examination of polyphenolic compounds against the SARS-CoV-2 virus M^pro^ protease revealed that SH6 had the best result of all tested compounds in the in silico model [80]. Moreover, the study performed by S. Luo et al. concerned the verification of bacterial neuraminidase inhibitory properties by nine compounds isolated from mock strawberry (*Duchesnea indica* Andr.). SH4 exhibited significant inhibitory activity in an in vitro model, which offers potential for its use as a new antiviral substance [20].

### 5.6. Anticancer Activity

Additionally studied features of sanguiins are their potential anticancer activity. Several investigations on SH6 have explained its anticancer effect due to its promising competency in inhibiting DNA topoisomerases I and II. Moreover, the compound acted as a blocker to HeLa cells. It inhibited their growth at an effective dose of 12 µM and also had a dose-dependent effect on intracellular topoisomerase activity. SH6 also exhibited significant antiangiogenic potential [82]. A study by Lee S. et al. on HT1080 human fibrosarcoma cells showed that this compound blocked KDR/Flk-1-Fc binding to VEGF165 in a dose-dependent manner. Moreover, the compound obstructed the VEGF-induced proliferation of HUVEC cells (IC_50_ ca. = 7.4 µg/mL) but was not active against HT1080 human fibrosarcoma cells [83].

The potential antitumor properties of sanguiins were also tested on PRMI-7951 melanoma cells. A moderate selective cytotoxicity was shown by SH2, SH6, and SH11 with ED_50_ results of 0.44, 0.5, and 5.0 µg/mL, respectively [68]. Furthermore, anticancer activity was tested with SH4 isolated from *Terminalia calamansanai* leaves against large tumor cells lines, including human promyelocytic leukemia HL-60 cells. The compound induced a decrease in human poly (ADP-ribose) polymerase [79] (PARP) associated with the cleavage of procaspase-3 and exhibited strong activation of proapoptotic caspase-3 in HL-60 cells. It is worth mentioning that SH4 does not affect healthy cells, suggesting this compound is selective against cancer cells [24]. In another examination, SH6 was responsible for modulating the Smad 2/3 signaling pathway by TGF-β1, increasing the expression of the epithelial marker E-cadherin, repressing the expression of Snail and the mesenchymal marker N-cadherin during TGF-β1-induced EMT (epithelial-mesenchymal transition), and regulating the expression of EMT-dependent genes induced by TGF-β1. In summary, SH6 inhibits the migration and invasion of A549 lung cancer in vitro by inhibiting TGF-β1 induction of EMT [84].

Moreover, SH6 showed a large number of antiproliferative, antimigration, and cytotoxic effects against human breast carcinoma cells. A study performed by Berdowska et al. proved that the tested compound exhibited an inhibitory effect on adriamycin-resistant cells (MCF-7/Adr) [85]. It also showed antimetastatic properties in MDA-MB-231 cells by reducing the expression of vascular endothelial growth factor (VEGF), phosphorylated Akt, and kinase 1/2 (ERK1/2) regulated by extracellular signals [86]. In addition, SH6 increased the ratio of Bax to Bcl-2 in both MCF-7 and MDA-MB-231 cells [79].

SH6 was also studied for its activity against A2780 human ovarian carcinoma cells. The tested compound induced an antiproliferative effect and a morphological change similar to apoptotic cell death but did not arrest the cancer cell cycle. Moreover, SH6 showed an early apoptotic effect, caspase activation, PARP cleavage, activation of mitogen-activated protein kinases (MAPKs), especially p38, and an increase in truncated p15/BID [87].

### 5.7. Estrogenic Activity

SH6 has also been tested for estrogenic activity against MCF-7 human breast cancer cells. The E-screen examination and the molecular docking analysis showed that the SH6 from *Rubus coreanus* exhibited the best binding energy of −250,149 kcal/mol. Additionally, at 100 μg/mL, *R. coreanus* extract significantly stimulated cell proliferation (574.57% ± 8.56%). The study results indicated that SH6 contributed to the estrogenic activity of *R. coreanus* by activating the ERα coactivator binding site [81].

### 5.8. Neuroprotective Activity

*Rubus* L. subgenus *R. watson*, *R. brigantinus*, and *R. vagabundus* extracts containing SH2, SH6, and SH10 were tested for their potential neuroprotective properties against SK-N-MC neuroblastoma cells. All digested extracts after 2 and 24 h of preincubation reduced basal ROS production. *Rubus brigantinus* and *R. vagabundus* extracts increased the mitochondrial transmembrane potential and the integrity of the cell membrane. Moreover, the extracts increased GSH levels while not changing the GSH/GSSG ratio. It is worth mentioning that there is insufficient evidence for the interaction of brain endothelial cells with polyphenol metabolites, which makes it difficult to determine the level of the passage of the compound across the blood–brain barrier [62].

### 5.9. Clinical Trials

As mentioned above, the efficacy of sanguiins is mainly limited to preclinical studies. However, there has been some research on black raspberry and pomegranate food products in clinical trials. Considering the fact that these products are rich in ellagitannins, it can be concluded that the biological activity may also be connected with the occurrence of sanguiins in the juice from berries and pomegranate. Nevertheless, there is a lack of information on clinical studies that use only sanguiins in medical treatment [44,94,95].

## 6. Pharmacokinetics of Sanguiins

Sanguiins, belonging to the ellagitannin group, show similar pharmacokinetics. In vitro studies have shown that ellagitannins are stable in the gastric environment, and in the presence of gastric enzymes, they are not hydrolyzed to ellagic acid. In addition, the absorption of ellagitannins in the stomach is impracticable due to their complex chemical structure. However, free ellagic acid molecules can be absorbed in the stomach. On the other hand, the intestinal environment, together with the gastrointestinal microbiota, creates suitable conditions for their hydrolysis and decomposition into urolithins and their derivatives, which pass through the intestinal wall into the enterohepatic circulation [96]. In addition, in vivo studies have shown that the metabolism of SH6 and SH10 in the liver is partly based on conjugation with glucuronic acid and sulfuric acid, leading to the formation of compounds such as urolithin A-*O*-glucuronide, urolithin A-sulfate, and urolithin B-3-*O*-glucuronide. Moreover, urolithins were detected in the unconjugated form. Conjugation of derivatives occurs at different rates and intensities; T_max_ of plasma urolithin glucuronides and sulfates is achieved in the vast majority of compounds 24 h after administration. Ultimately, conjugated and unconjugated compounds are excreted in the urine at varying intervals, up to 48 h after ingestion. Further in vivo clinical studies linked to full pharmacokinetic analysis are necessary to fully determine the participation of urolithins in the therapeutic effects of ellagitannin-rich plants [3,97,98].

## 7. Conclusions

The isolation and structure determination, accompanied by the measurement of the diverse pharmacological activities of each isolated sanguiin, has brought about a marked change in the concept of these compounds as active components of medicinal plants. In summary, sanguiins, especially sanguiin H-6, show evidence of promising action in various biological contexts, particularly in respect of their anticancer, antiradical, and antiviral properties. Apart from that, further studies involving drug delivery may improve the effectiveness of these compounds toward the drug target sites. Furthermore, it is worth considering performing a supplementary survey on their metabolism and toxicology patterns with molecular docking and molecular dynamics simulation to understand their mechanisms of action fully.

## Figures and Tables

**Table 1:** Plants containing sanguiin and its traditional uses.

Plant	Family	Geographical Location	Type of SH	Amount of SH	Traditional Medicine Uses	References
*Alchemilla vulgaris*	Rosaceae	Germany	SH6 SH10 isomers	not given	wounds, eczema, and inflamed mucosa	[19]
*Alchemilla mollis*	Rosaceae	Germany	SH6 SH10 isomers	not given	wounds, eczema, and inflamed mucosa	[19]
*Duchesnea indica*	Rosaceae	China	SH4	0.0046 mg/g of dried fruits	fever, inflammation, cancer	[20,30]
*Fragaria vesca*	Rosaceae	Italy	SH6	not given	inflammation-related diseases	[22,31]
*Fragaria ananassa*	Rosaceae	Italy	SH6	not given	not found	[22]
*Rosa laevigata*	Rosaceae	China	SH4	0.03 mg/g of dried pecarps	urinary incontinence, diarrhea, pain, burns, toothache	[32]
*Rubus aleaefolius*	Rosaceae	China	SH2 ethyl ester	0.028 mg/g of dried roots	various types of hepatitis	[33,34]
*Rubus adenotrichus*	Rosaceae	Costa Rica, Trinidad Ecuador	SH6	4.2 mg/g of dried berries	not found	[35,36]
*Rubus arcticus*	Rosaceae	propagated vegetatively	SH5 SH6 SH10	not given	not found	[37]
*Rubus ulmifolius*	Rosaceae	Portugal	SH10 isomer	not given	diarrhea, menstrual pain, menopause disorders, liver diseases, aphtha, gingivitis	[38]
*Rubus chamaemorus*	Rosaceae	Finland	SH6 SH10 isomers	not given	scurvy and diarrhea	[39,40,41]
*Rubus caesius*	Rosaceae	Poland	SH6	5.79 mg/g of dried leaves	uterine relaxant, stimulant during confinement, diarrhea and similar enteric disorders, an astringent	[16,42,43]
*Rubus hirsutus*	Rosaceae	Japan	SH6 SH11	73.92 mg/g of dried leaves	not found	[18]
				not given		
*Rubus occidentalis*	Rosaceae	Poland	SH6	10.78–50.45 mg/g of plant extract from shoots	common cold, fever and flu-like infections, management of impotence, spermatorrhea, enuresis, asthma, allergic diseases	[27,44,45,46]
*Rubus lambertianus*	Rosaceae	Taiwan, Japan	SH2 SH6 SH11	not given	not found	[18,25]
*Rubus parvifolius*	Rosaceae	Japan	SH2 SH6 SH11	not given	fever, angina, enteritis, hepatitis, concretion, eczema, rheumatism	[18,25,47]
*Rubus crataegifolius*	Rosaceae	Japan	SH2 SH6 SH11	not given	diabetes mellitus	[18,25,48]
*Rubus pedatus*	Rosaceae	Japan	SH6 SH11	not given	not found	[18]
*Rubus palmatus*	Rosaceae	Japan	SH2 SH6 SH11	not given	not found	[18,25]
*Rubus chingii*	Rosaceae	Japan	SH2 SH6 SH11	not given	invigorating Qi, losing weight, blackening hair, tonifying kidney, enriching essence, impotence	[18,25,49,50]
*Rubus sieboldii*	Rosaceae	Japan	SH2	not given	not found	[25]
*Rubus corchorifolius*	Rosaceae	Japan	SH2	not given	impotence, seminal emission	[25,51]
*Rubus palmatus* var. *coptophyllus*	Rosaceae	Japan	SH2	not given	not found	[25]
*Rubus idaeus*	Rosaceae	Japan Poland Italy	SH2	not given	enterocolitis, bronchitis, prostate disorders, analgesic, cold, cough, fever	[25,27,52,53]
			SH6	1.7–6.33 mg/g of plant extract from shoots		
*Rubus mesogeanus*	Rosaceae	Japan	SH2	not given	not found	[25]
*Rubus calycinoides*	Rosaceae	Taiwan	SH2	not given	not found	[25]
*Rubus phoenicolasius*	Rosaceae	Japan	SH2 SH6 SH11	not given	rheumatism, irregular menstruation, kidney ailments	[18,25,54]
*Rubus loganbaccus* x *Rubus baileyanus*	Rosaceae	New Zealand	SH2 SH6 SH10	not given	not found	[55]
*Rubus glaucus*	Rosaceae	Trinidad, Costa Rica, Ecuador	SH6	2.45 mg/g of dried berries	diarrhea, wounds, burns	[17,35]
*Rubus coreanus*	Rosaceae	Korea, Japan	SH2 SH5 SH4 SH6 SH11	not given	impotence, pollution, premature ejaculation, frequency of urination	[18,56,57,58,59,60]
*Rubus fruticosus*	Rosaceae	Poland, Japan	SH6	1.35–5.47 mg/g of dried berries	dysentery, diarrhea, whooping cough, colitis, toothache, pain	[18,26,61]
			SH11	not given		
			SH2 isomer	not given		
*Rubus irirasem*	Rosaceae	Japan	SH6 SH11	not given	not found	[18]
*Rubus hiraseanus*	Rosaceae	Japan	SH6 SH11	not given	not found	[18]
*Rubus vagabundus*	Rosaceae	Portugal	SH2 SH6 SH10	not given	not found	[62]
*Rubus brigantinus*	Rosaceae	Portugal	SH2 SH6 SH10	not given	not found	[62]
*Rubus radula*	Rosaceae	Poland	SH6	16.66 mg/g of dried leaves	not found	[43]
*Rubus montanus*	Rosaceae	Poland	SH6	16.95 mg/g of dried leaves	not found	[43]
*Rubus gracilis*	Rosaceae	Poland	SH6	18.07 mg/g of dried leaves	not found	[43]
*Rubus macrophyllus*	Rosaceae	Poland	SH6	14.48 mg/g of dried leaves	not found	[43]
*Rubus pericrispatus*	Rosaceae	Poland	SH6	14.49 mg/g of dried leaves	not found	[43]
*Rubus subcatus*	Rosaceae	Poland	SH6	59.79 mg/g of dried leaves	not found	[43]
*Rubus ambrosius*	Rosaceae	Poland	SH6	21.11 mg/g of dried leaves	not found	[43]
*Rubus fasciculatus*	Rosaceae	Poland	SH6	23.24 mg/g of dried leaves	not found	[43]
*Rubus nessensis*	Rosaceae	Poland	SH6	12.22 mg/g of dried leaves	not found	[43]
*Rubus glivicensis*	Rosaceae	Poland	SH6	48.46 mg/g of dried leaves	not found	[43]
*Rubus bifronus*	Rosaceae	Poland	SH6	39.48 mg/g of dried leaves	not found	[43]
*Rubus praecox*	Rosaceae	Poland	SH6	18.49 mg/g of dried leaves	not found	[43]
*Rubus perrobustus*	Rosaceae	Poland	SH6	53.02 mg/g of dried leaves	not found	[43]
*Rubus parthenocissus*	Rosaceae	Poland	SH6	11.41 mg/g of dried leaves	not found	[43]
*Rubus pseudidaeus*	Rosaceae	Poland	SH6	15.07 mg/g of dried leaves	not found	[43]
*Rubus constrictus*	Rosaceae	Poland	SH6	24.38 mg/g of dried leaves	not found	[43]
*Rubus wimmerianus*	Rosaceae	Poland	SH6	64.44 mg/g of dried leaves	not found	[43]
*Rubus orthostachys*	Rosaceae	Poland	SH6	45.60 mg/g of dried leaves	not found	[43]
*Rubus plicatus*	Rosaceae	Poland	SH6	58.48 mg/g of dried leaves	not found	[43]
*Rubus pedemontanus*	Rosaceae	Poland	SH6	63.51 mg/g of dried leaves	not found	[43]
*Rubus grabowski*	Rosaceae	Poland	SH6	49.77 mg/g of dried leaves	not found	[43]
*Sanguisorba tenuifolia* var. *parviflora*	Rosaceae	Japan	SH2 SH11	not given	not found	[25]
*Sanguisorba officinalis*	Rosaceae	Japan	SH1	not given	leukopenia, hemorrhaging, burns	[13,25,63,64]
			SH2	not given		
			SH3	not given		
			SH6	1.6 mg/g of dried leaves		
			SH11	not given		
*Sanguisorba tenuijolia* var. *alba*	Rosaceae	Japan	SH6 SH11	not given	not found	[18]
*Punica granatum*	Lythraceae	Spain	SH10 isomers	not given	inflammation, rheumatism, pain, snakebites, diabetes, burns, leprosy, vermifugal and taenicidal agent	[23,65]
*Euphorbia* *fischeriana*	Euphorbiaceae	China	SH5	0.072 mg/g of dried roots	dyspepsia, abdominal distension, abdominal pain, cough, external applications as a cure for scabies and tuberculosis of lymph nodes	[21,66]
*Terminalia* *calamansanai*	Combretaceae	Taiwan	SH4	0.098 mg/g of dried leaves	lithotriptic	[24]

**Table 2:** Chromatographic techniques for the analysis of sanguiins.

Compound	Stationary Phase/Column	Mobile Phase	Conditions (Flow Rate, Injection Volume)	Detection	References
SH6, SH10 isomers	SunFire C18 RP	1% FA and ACN/H_2_O (9:1, *v/v*)	0.21 mL/min; 5 μL	280 nm	[19]
SH4	Phenomenex Gemini C18; Waters Symmetry C18; Phenomenex Kinetex C18; Phenomenex Luna C18	1% FA and MeOH	1–15 mL/min	310 nm	[20]
	Toyopearl HW-40F	70% MeOH	-	-	[32]
	LiChroprep RP C18	0.05% TFA and CH_3_CN (95:5)	1 mL/min	280 nm	[24]
SH2, ethyl ester	ODS	MeOH–H_2_O (35:65)	-	-	[33]
SH6	Lichrospher ODS-2 RP	2% FA and ACN/H_2_O/FA (80:18:2, *v*/*v*/*v*)	0.5 mL/min; 10 μL	200–600 nm	[35]
	Discovery HS C18	0.1% TFA and 0.1% TFA in a mixture of H_2_O:ACN (50:50 *v/v*)	0.3 mL/min; 1 μL	520 nm	[27]
	Fuji-gel ODS-G3	MeOH–H_2_O (7:3)	-	-	[25]
	UPLC BEH C18	4.5% FA and ACN	0.45 mL/min; 10 μL	240 nm	[43]
SH5, SH6, SH10	ODS Hypersil	ACN and 1% FA	2 mL/min; 15 μL	280 nm	[37]
SH10 isomer	Spherisorb S3 ODS-2 C18	1% FA and ACN	0.5 mL/min;	280 nm	[38]
	BlueOrchid C18; Hypersil Gold C18; Kinetex PFP	ACN + 1% FA and H_2_O	0.2 mL/min; 5 μL	-	[23]
SH2	MCI-gel CHP 20P	mixture of MeOH and H_2_O	-	-	[25]
SH5	Sephadex LH-20	mixture of MeOH and H_2_O	-	-	[21]
SH6, SH11	Superspher Si 60	hexane-MeOH-THF-HCO_2_H + (COH)_2_O	1.5 mL/min;	280 nm	[18]
SH2, SH6, SH10	Synergy Hydro RP C18	ACN:H_2_O	10 mL/min; 50–200 μL	280 nm	[55,62]

**Table 3:** Bioactivities of sanguiins reported in in vitro and in vivo experimental models.

Activity	Experimental Model	Exposure	Concentration	Efficacy	References
Anti-inflammatory	Rat neutrophils	60 min chemotaxis and 2 h toxicity in in vitro assays	0, 1, 2.5, 5, and 10 μM SH11, SH6, and SH2	IC_50_ of SH2, SH6 and SH11 of inhibitory activity on CINC-1-dependent neutrophil chemotaxis was about: 10, 4, and 2.5 μM, respectively 95% of the cells were living after 2 h-incubation with sanguiins	[70]
	Human AGS gastric epithelial cells	1 h for NF-κB nuclear translocation, 6 h for NF-κB-driven transcription, and 6 h for IL-8 release in in vitro assays	0.25–10 μM SH6	IC_50_: 0.87 ± 0.16 µM—without stimulation and 1.9 ± 0.23 µM with IL-1β IC_50_: 1.5 ± 0.35 µM—TNFα stimulated and 2.7 ± 0.30 µM—IL-1β stimulated At 2.5 µM SH6 completely inhibited release of IL-8 with IC_50_: 0.58 ± 0.05 μM—TNFα-induced and 1.03 ± 0.06 μM—IL-1β-induced	[71]
Antioxidant	Male LWH Wistar rats	In vivo, rats were fed orally with SH6 for 30 days	10 mg/kg body weight/day	Level of 3-nitrotyrosine in plasma reduced from 607.6 ± 15.6 to 294.8 ± 26.1 pmol/mL TBA-reactive substance decreased from 1.31 ± 0.30 to 0.83 ± 0.14 nmol/mg protein GSH level increased from 1.44 ± 0.25 to 2.44 ± 0.26 nmol/mg (sham treatment—3.35 ± 0.25) Glutathione peroxidase level increased from 107.6 ± 5.2 to 115.6 ± 6.0 U/mg (sham treatment—141.3 ± 16.0) DNA fragmentation level decreased from 23.4% ± 2.0% to 16.9% ± 1.6% Caspase-3 decreased from 8.26 ± 0.71 to 5.95 ± 0.36 pmol AMC/mg protein/min Urea nitrogen decreased from 75.2 ± 3.1 to 59.5 ± 2.3 mg/dL Cr decreased from 1.84 ± 0.13 to 1.34 ± 0.12 mg/dL	[72]
	Fremy’s salt	20 min electron spin resonance spectroscopy in situ assay	extracts diluted to 5% (*v/v*) with ethanol and water (12:88, *v/v*); 1.0 mL portion	1.7 × 10^17^ per gram f.w. Fremy’s radicals reduced by SH6	[73]
	HT22 murine hippocampal cells	8 h in vitro assay	0, 10, and 20 μM SH11	Intracellular ROS: viability of cells I creased at a concentration: 20 µM (glutamine present), 10 µM (glutamine absent), and 20 µM (glutamine absent). At a 10 µM with glutamine present observed slight decrease in viability	[74]
	DPPH, methyl linoleate and diene hydroperoxide	15 min, 72 h, and 2 h in situ assays	2, 5, 10, 50, and 250 μM of raspberry ET dimers and trimers	DPPH test (ROS %): 2 μM: raspberry ET dimers and trimers: 20 ± 0.4; cloudberry ET dimers and trimers: 21 ± 0.1 5 μM: raspberry ET dimers and trimers: 40 ± 0.1; cloudberry ET dimers and trimers: 47 ± 0.2 10 μM: raspberry ET dimers and trimers: 79 ± 0.3; cloudberry ET dimers and trimers: 74 ± 1.7 Methyl linoleate: inhibition % 50 μM; raspberry ET dimers and trimers: 24 ± 4.9; cloudberry ET dimers and trimers: 21 ± 4.9 100 μM: raspberry ET dimers and trimers: 37 ± 0.0; cloudberry ET dimers and trimers: 13 ± 3.2 250 μM: raspberry ET dimers and trimers: 37 ± 3.2; cloudberry ET dimers and trimers: 59 ± 3.2 Emulsion: inhibition (%) of conjugate diene hydroperoxide formation: 50 μM: raspberry ET dimers and trimers: 90 ± 0.7; cloudberry ET dimers and trimers: 91 ± 0.0 250 μM: raspberry ET dimers and trimers: 96 ± 0.4; cloudberry ET dimers and trimers: 95 ± 0.0	[75]
	ABTS and FRAP assays	6 min in situ ABTS assay, 8 min in situ FRAP assay	not given	ABTS radical scavenging (mmol TE/g dm): *R. pedemontanus*—212.69 and *R. parthenocisus*—c.a. 203 FRAP ability: *R. pedemontanus*—192.91 and *R. parthenocissus*—192.53	[43]
	mice macrophage and sodium nitroprusside	24 in vitro macrophage incubation, 150 min in situ sodium nitroprusside assay	0, 12.5, 25, and 50 μM of SH6 in macrophage assay, 0, 2.5, 5, 12.5, 25, 50, and 100 μM of SH6 in sodium nitroprusside assay	Macrophage’s assay (µM): Nitrite level reduced above 50% at concentrations 12.5, 25, and 50 Cell viability (%) increased at concentrations 12.5, 25, and 50 The enzymatic activity of iNOS (pmol/mg protein/min) was: 12.5 µM SH6-19.98; 25 µM SH6—9.80; 50 µM SH6—7.01 Decreased NO generation from sodium nitroprusside: 0 μM: 13.15 ± 0.11; (2.5 μM): 8.29 ± 0.07; 5 μM: 8.16 ± 0.09; 12.5 μM: 8.07 ± 0.10; 25 μM: 7.69 ± 0.07; 50 μM: 6.91 ± 0.10; 100 μM: 4.78 ± 0.05	[76]
Osteoclastogenesis inhibitory	8-week-old male C57BL/6J mice	intraperitoneal injections for 5 days	10 μg/body weight(g)/day of SH6	Mice treated with both TNF-α and SH6—TRAP-positive amount of osteoclasts significantly reduced and the percentage of ES/BS (eroded surface/bone surface)	[47]
	bone marrow macrophages (BMMs)	72 h in vitro assay	0, 1, 5, 10, and 25 μM of SH6	SH6 at concentrations >5 μM downregulated the expression of NFATc1 and its target proteins, c-Src, and cathepsin K	
	RAW-D cells	72 h in vitro assay	5 μM of SH6	SH6 strongly inhibited the nuclear translocation of NFATc1, phosphorylated-c-Fos, and NF-κB	
	BMMs and RAW-D cells	72 h in vitro assay	0–50 μM of SH6 in BMM and RAW-D cells assays	Dose-dependent inhibition of multinucleated osteoclast formation in BMM cells; cytotoxicity was observed at 25 and 50 μM. The number of TRAP-positive RAW-D-derived osteoclasts decreased significantly after treatment with >0.1 μM SH6; cytotoxicity was observed at >10 μM SH6	
Antibacterial	*Streptococcus group A, B, C* *S. pneumoniae* *E. faecalis* *C. diphtheriae* *B. subtilis* *C. sporogenes* *S. aureus* *S. epidermidis* *N. meningitidis* *M. catarrhalis* *H. influenzae* *H. pylori* *K. pneumoniae*	48 h in vitro assay	SH6 concentrations: geometric progression from 0.015 to 1 mg/mL	MIC (mg/mL): *Streptococcus* group A: 0.5 *S. pneumoniae*: 0.5, *C. diphtheriae*: 0.03 *B. subtilis*: 0.5 *C. sporogenes*: 0.06 *S. aureus*: 0.25 *S. epidermidis*: 0.125 *M. catarrhalis*: 0.5 2. MBC (mg/mL): *Streptococcus* group A: 0.5 *S. pneumoniae*: 0.5 *C. diphtheriae*: 0.03 *S. epidermidis*: 0.125	[27]
	*C. perfringens* *E. coli* *L. plantarum* *S. aureus*	24 h in vitro incubation	0.5 mM of SH6	*S. aureus* inhibition: reduction in the growth from 10^9^ CFU/mL to 10^3^ CFU/mL *E. coli* inhibition: reduction in the growth from 10^9^ CFU/mL to 10^7^ CFU/mL *3.L. plantarum* inhibition: reduction in the growth from 8.0 × 10^8^ CFU/mL to 6.0 × 10^8^ CFU/mL *4.C. perfringens* inhibition: reduction in the growth from 7.0 × 10^8^ CFU/mL to 2.0 × 10^8^ CFU/mL	[77]
	*E. coli, E. faecalis* *K. pneumoniae*, *M. morganii*, *P. mirabilis*, *P. aeruginosa*, *L. monocytogenes*, *MRSA*, *MSSA*	not given	100 mg/mL (stock solution) *R. ulmifolius* extract; SH10: 9.6 ± 0.1 mg/g	MIC: E. coli, M. morganii, E. faecalis *L. monocytogenes, MSSA:* 5 mg/mL *Proteus mirabilis, MRSA:* 10 mg/mL *P. aeruginosa, K. pneumoniae:* >20 mg/mL	[78]
Antifungal	*C. albicans*	not given	100 mg/mL (stock solution) *R. ulmifolius* extract; SH10: 9.6 ± 0.1 mg/g	MIC: 5 mg/mL	
Antiviral	NA from *C. perfringens*	30 min in situ assay	SH4 solution	Inhibitory activity of SH4 on NA from *Clostridium perfringens:* IC_50_ (μmol/L): 17.48 ± 2.9	[20]
	spike glycoprotein of SARS-CoV-2 virus	in silico molecular docking assay	SH6 and SH2 molecular structures	SH6: docking score of—9.8 kcal/mol SH2: docking score of—8.7 kcal/mol	[79]
	M^pro^ protease and spike glycoprotein of SARS-CoV-2 virus	in silico molecular docking assay	SH6 molecular structure	M^pro^ protease docking score: −10.3 kcal/mol Spike glycoprotein docking score: −9.8 kcal/mol	[80]
Estrogenic	MCF-7 human breast adenocarcinoma cell	144 h in vitro proliferation assay	SH6 at 0, 25, 50 and 100 μM, *Rubus coreanus*: 0, 5, 10, 25, 50, and 100 μg/mL	SH6: 127.41% ± 0.26% cell proliferation at 100 μM; *R. coreanus*: 574.57% ± 8.56% cell proliferation at 100 μg/mL	[81]
	Estrogen Receptor α	in silico molecular docking assay	SH6 molecular structure	SH6: docking score of—250.149 kcal/mol	
Neuroprotective	SK-N-MC neuroblastoma cells	2 and 24 h in vitro assay	commercial blackberry and *R*. *vagabundus*: 0, 0.25, 0.5, and 1 µg GAE/mL, *R. brigantinus*: 0, 0.1, 0.2, and 0.4 µg GAE/mL	All blackberry digested extracts at 2 and 24 h preincubation reduced basal ROS production. Under oxidative stress conditions, blackberry extracts did not reduce ROS production above 20% The best activity (20%) exhibited *R. brigantinus* extract with a concentration of 0.4 µg GAE/mL)	[62]
		24 h in vitro assay	Commercial blackberry and *R. vagabundus*: 0, 0.25, 0.5, and 1 µg GAE/mL, *R. brigantinus*: 0, 0.1, 0.2, and 0.4 µg GAE/mL	*R. brigantinus* and *R. vagabundus* extracts simultaneously increased mitochondrial transmembrane potential and cell membrane integrity Preincubation with the IN fractions from *R. brigantinus* and *R. vagabundus*, although not changing GSH/GSSG ratio, increased GSH levels	
Anticancer	HeLa cells	72 h in vitro assay	Cytotoxicity: 0–25 µM of SH6 DNA cleavage: 10, 15, and 25 µM	Growth inhibitory effects of SH2 against HeLa cells occurred over a narrow dose range, with an ED_50_ of 12 µM SH6 interfered with drug-stimulated DNA break formation in a dose-dependent fashion. This effect was quite similar against both DNA topoisomerases with IC_50_ values of ~15 µM	[82]
	Topoisomerase I and II	30 min in situ assay	Topoisomerase I: 0, 19, 38, and 75 nM of SH6 Topoisomerase II: 0, 0.05, 0.1 0.2, 0.4, and 0.8 µM of SH6	SH6 interfered with topoisomerase I-mediated DNA cleavage: IC_50_ value = 0.02 µM Topoisomerase Il-dependent DNA cleavage of linear DNA induced by the inhibitor VP-16 was prevented by simultaneous exposure to SH6. IC_50_ value = 0.l6 µM	
	Topoisomerase I and II	30 min in situ assay	0, 0.1, 0.2, 0.4, 0.6, 1.2, and 2.4 µM of SH6	Reaction of topoisomerase I-dependent DNA relaxation with IC_50_ value = 1 µM Topoisomerase II was completely inhibited at 0.5 µM of SH6. IC_50_ = 0.01 µM Relative potency of SH6 was 100-fold greater for topoisomerase II than for I	
	HUVECs and HT1080 cells	72 h in vitro XTT incorporation assay	SH6: concentrations up to 20 µg/mL	SH6 efficiently blocked the VEGF-induced HUVEC proliferation in a dose-dependent manner (IC_50_ = 7.4 µg/mL)	[83]
	PRMI-7951 melanoma cells	in vitro cytotoxicity assay	SH2, SH6, and SH11 solutions	ED_50_ against melanoma RPMI-795 l: SH2: 0.44 µg/mL SH6: 5.00 µg/mL SH11: 0.50 µg/mL	[68]
	HL-60 and PBMCs	12 h in vitro treatment	HL-60: 100 µM, PBMCs: 400 µM of SH4	Inhibition of cell growth: cell values: 93.0% ± 0.42% (HL-60) 45.6% ± 0.30% (PBMCs)	[24]
	AGS, HeLa, Hep G2, HT 29, and T 24 cell lines	24 h in vitro treatment	100 µM of SH4	Inhibition of cell growth: cell values 2.69% ± 2.44% (AGS) 24.34% ± 4.73% (HeLa) 38.99% ± 2.19% (Hep G2) 8.10% ± 6.37% (HT 29) 80.58% ± 5.98% (T 24)	
	HL-60 cells	12 h in vitro assay	serial dilution concentrations from 0 to 400 µM of SH4	Cytotoxic effect of SH4 was more pronounced in the leukemia HL-60 cells than in the normal PBMCs	
			25, 50, and 100 µM of SH4	SH4 showed significantly inhibited DNA fragmentation in a dose-dependent manner	
			100 µM of SH4	Treatment with SH4 showed a decrease in the 116 kDa PARP and a dose-dependent increase in inactive PARP	
			50 and 100 µM of SH4	SH4 showed a significant activation of caspase-3 in HL-60 in dose-dependent manner	
	A549 lung cancer cells	48 h in vitro assay	5 and 10 µM of SH6	SH6 blocked the migration and invasion capabilities of the A549 cells during TGF-β1 induction of the EMT	[84]
		48 h in vitro assay	5 and 10 µM of SH6	Significant decreases in the expression levels of nine genes	
		2 h in vitro pretreatment	5 and 10 µM of SH6	Snail expression was decreased by SH6 treatment in a dose-dependent manner. Plasminogen activator inhibitor type-1 (PAI-1) expression decreased after SH6 treatment in a dose-dependent manner	
			5 and 10 µM of SH6	SH6 antagonizes the phosphorylation of Smad2 and Smad3	
			5 and 10 µM of SH6	TGF-β1 induction of the mesenchymal phenotype was inhibited	
		48 h in vitro assay	1, 2.5, 5, 10, 25, 50, 75, and 100 µM of SH6	Concentrations of SH6 ≤ 25 µM did not affect the proliferation of A549 cells. Proliferation of A549 cells was inhibited with ≥ 50 µM	
	MCF-7/Adr and MCF-7/wt cells	48 h in vitro incubation; MTT assay	10, 20, 40, 79, 157, and 313 µM of SH6	SH6 inhibited the viability of MCF-7/Adr cell line within the whole concentration range. (EC_50_ = 38 µM). SH6 caused fluctuations around the 100% control viability of MCF-7/wt cells	[85]
	MDA-MB-231 human breast cancer cells	24 h in vitro assay	0 and 6.25 µM of SH6	SH6 decreased the protein expression of VEGF, phosphorylated Akt, and ERK1/2	
			0, 6.25, 12.5, 25, 50, 100, and 200 µM of SH6	Treatment with up to 25 µM had no effect on MDA-MB-231 cells. Treatment with 200 µM decreased cell viability	
	HUVECs		0 and 6.25 µM of SH6	The percentage inhibition of migration of 6.25 µM SH6-treated cells was 37.6% of that observed in the control group. SH6 at a concentration of 6.25 µM significantly blocked tube formation (41.5% of control)	[86]
			0, 6.25, 12.5, 25, 50, 100, and 200 µM of SH6	12.5 µM with no effect on the HUVECs. Treatment with 25 to 200 µM decreased cell viability	
	MCF-7 and MDA-MB-231 cells	24 h in vitro assay	0, 50, and 100 µM of SH6 for MCF-7 and MDA-MB-231 cells	SH6 increased Bax expression in MCF-7 cells SH6 decreased Bcl-2 expression in MDA-MB-231 cells	[79]
			50 and 100 µM of SH6	SH6 increased the cleavage of caspase-8, caspase-3, and PARP, but not that of caspase-9 in MCF-7 cells. SH6 increased the cleavage of caspase-8, caspase-9, and caspase-3, as well as that of PARP in MDA-MB-231 cells	
			0, 5, 10, 25, 50, and 100 µM of SH6	SH6 at a concentration of 100 µM for MCF-7 and MDA-MB-231 significantly reduced viabilities to approximately 69% and 63%, respectively. SH6 reduced the viabilities of both cell lines in a concentration-dependent manner	
	A2780 human ovarian carcinoma cells	24 h in vitro assay	0, 10, 20, and 40 µM of SH6	Increasing amount of: cleaved caspase-8, cleaved caspase-3, tBID cleaved RARP, and p-p38 with increasing SH6 dose	[87]
			20 and 40 µM of SH6	Treatment of A2780 cells with SH6 induced an increase in the fraction (Annexin V+/PI-) of early apoptotic cells from 4.17% to 41.76%	
			0, 10, 20, and 40 µM of SH6	Treatment of A2780 cells with SH6 induced a decrease in cell viability in a dose-dependent manner	

## Data Availability

Data are contained within the article.

## Abbreviations

SH2	sanguiin H-2
SH3	sanguiin H-3
SH5	sanguiin H-5
SH6	sanguiin H-6
SH10	sanguiin H-10
SH11	sanguiin H-11
ADMET	absorption, distribution, metabolism, excretion, toxicity
CINC-1	cytokine-induced neutrophil chemoattractant
TNFα	tumor necrosis factor α
IL-1β	interleukin-1β
d.w.	dry weight
ONOO-	peroxynitrite
LPS	lipopolysaccharide
NFATc1	nuclear factor of activated T cells 1
NF-κB	nuclear factor-κB
c-Src	proto-oncogene tyrosine-protein kinase Src
TNF-α	tumor necrosis factor
PARP	poly (ADP-ribose) polymerase
EMT	epithelial-mesenchymal transition
TGF-β1	transforming growth factor beta 1
VEGF	vascular endothelial growth factor
iNOS	inducible NO synthase
ETs	ellagitannins
DPPH	2,2-diphenyl-1-picrylhydrazyl
ROS	reactive oxygen species
ABTS	2,29-azinobis-(3-ethylbenzo-thiazoline-6-sulfonic acid)
FRAP	ferric reducing ability of plasma
MRSA	methicillin-resistant *Staphylococcus aureus*
MSSA	methicillin-sensitive *Staphylococcus aureus*
MIC	minimum inhibitory concentration;
MBC	minimum bactericidal concentration
GSH	glutathione
GSSG	glutathione disulfide
BMMs	bone marrow macrophages
tBID	truncated BID
HUVECs	human umbilical vein endothelial cells
MCF-7/wt	MCF-7 human breast cancer cell (wild type)
FA	formic acid
TBA	thiobarbituric acid
ACN	acetonitrile
AMC	acceptable means of compliance
iNOS	inducible nitric oxide synthase
TRAP	tartrate-resistant acid phosphatase
ES/BS	eroded surface/bone surface
BMM	bone marrow-derived macrophages
NFATC1 T_max_	nuclear factor of activated T cells 1 time take to reach maximum concentration
